# Wildlife Reservoir for Hepatitis E Virus, Southwestern France

**DOI:** 10.3201/eid2107.141909

**Published:** 2015-07

**Authors:** Sebastien Lhomme, Sokunthea Top, Stephane Bertagnoli, Martine Dubois, Jean-Luc Guerin, Jacques Izopet

**Affiliations:** Institut National de la Santé et de la Recherche Médicale, Toulouse, France (S. Lhomme, S. Top, M. Dubois, J. Izopet);; Université Paul Sabatier, Toulouse (S. Lhomme, S. Top, M. Dubois, J. Izopet);; Le Centre Hospitalier Universitaire Purpan, Toulouse (S. Lhomme, M. Dubois, J. Izopet);; Institut National Polytechnique, Toulouse (S. Bertagnoli, J.-L. Guerin);; Institut National de la Recherche Agronomique, Toulouse (S. Bertagnoli, J.-L. Guerin)

**Keywords:** hepatitis E virus, reservoir, wildlife, viruses, France, hepatitis

## Abstract

Pigs are a reservoir for hepatitis E virus (HEV). To determine the relative contribution of game to the risk for human HEV infection in southwestern France, we tested wildlife samples. HEV RNA was in 3.3% of wildlife livers, indicating that in this region, eating game meat is as risky as eating pork.

Hepatitis E virus (HEV) is a causative agent of acute hepatitis worldwide. According to the Ninth Report of the International Committee on the Taxonomy of Viruses (http://ictvonline.org/), HEV is the sole member of the genus *Hepevirus* in the family *Hepeviridae*. HEV is a nonenveloped, single-stranded, positive-sense RNA virus containing ≈7.2 kb. Its genome contains 3 open reading frames (ORFs)—ORF1, ORF2, and ORF3—which encode nonstructural proteins, the capsid protein, and a small protein involved in virus egress, respectively (*1*).

Phylogenetic analysis of HEV sequences has led to the identification of 4 major genotypes (*1*). Genotypes 1 (HEV1) and 2 (HEV2) are pathogenic to humans only. HEV1 is present mainly in Asia and Africa, and HEV2 is in Africa and Mexico. In developing countries, HEV1 and HEV2 transmission is waterborne because of inadequate sanitary conditions. Genotypes 3 (HEV3) and 4 (HEV4) infect not only humans but also pigs, wild boars, deer, and other mammals. HEV3 is widespread, but HEV4 occurs mainly in Asia and was recently introduced into Europe (*1*). Pigs are a major reservoir of HEV3 and HEV4 (*2*); however, in recent years, the host range of HEV has expanded substantially (*3*).

HEV is hyperendemic to the Midi-Pyrénées area of southwestern France; annual incidence of cases among humans is 3.2% (*4*), and seroprevalence among blood donors has reached 52.5% (*5*). A multivariate analysis reported that the only factor associated with autochthonous HEV infection in this region was the consumption of game meat (*6*). However, the prevalence of HEV RNA in wildlife, especially wild boars and deer, has yet to be explored. Identifying the most commonly infected animals (sources of transmission) could help prevent zoonotic foodborne transmission. HEV strains have been recently identified in rabbits (*7*). Because HEV strains in rats have been recently described (*8*), we questioned the capacity of coypu to act as an HEV reservoir. Coypu are large, herbivorous, semiaquatic rodents that usually live in fresh or brackish water. In this study, we assessed the prevalence of HEV RNA among wild boars (*Sus scrofa*), deer (*Cervus elaphus*), rabbits (*Oryctolagus cuniculus*), and coypu (*Myocastor coypus*) and, thus, the potential for these animals to act as sources of HEV infection for persons living in the Midi-Pyrénées area.

## The Study

Samples of liver and bile were collected from 86 wild boars, 62 deer, 20 wild rabbits, and 78 coypu in the Midi-Pyrénées area. The wild boars and deer were hunted from February 2010 through January 2011, rabbits were hunted from October 2013 through February 2014, and coypu were hunted in April 2011. 

RNA was extracted from 30 mg of liver by using RNeasy Mini Kits or from 140 μL of bile by using QIAamp Viral RNA Mini Kits as specified by the manufacturer (QIAGEN, Courtaboeuf, France). Real-time PCR based on ORF3 was used to detect and quantify HEV RNA in plasma samples as previously described (*9*). The limit of detection was 100 copies/mL.

HEV RNA was detected in 5 (5.8%) wild boar livers, 2 (3.2%) deer livers, 1 (5.0%) wild rabbit liver, and no coypu livers (Table). Thus, the overall prevalence of HEV RNA among wildlife, irrespective of species, was 3.3% (8/246) (95% CI 1.1%–5.5%). Because bile samples were available only from animals with negative HEV RNA liver samples, no bile sample was positive for HEV RNA.

**Table:** Hepatitis E virus RNA among wildlife, southwestern France*

Source, dates collected	No. tested	No. (%) HEV RNA positive	Virus concentration, median log copies/g (range)
Wild boars, 2010–2011			
Liver	86	5 (5.8)	2.80 (1.57–8.05)
Bile	29	0	NA
Deer liver, 2010–2011	62	2 (3.2)	2.78 (1.11–3.07)
Wild rabbits, 2013–2014			
Liver	20	1 (5.0)	8.70
Bile	13	0	NA
Coypu liver, 2011	78	0	NA

*NA, not applicable.

## Conclusions

The overall prevalence of HEV RNA in game (3.3%) is similar to that among pigs. A recent nationwide study in France reported that HEV RNA was present in the livers of about 4% of farmed pigs of slaughter age (*10*). These contaminated livers can then enter the food chain and be responsible for foodborne zoonotic HEV infection. Such pork products, especially raw or undercooked liver, are a major source of exposure to the virus (*11*). HEV has been found in pig liver sausages (*5*,*12*,*13*). Our findings suggest that game meat from wild boar, deer, and rabbit contribute to HEV epidemiology in the Midi-Pyrénées area. However, residents of this area eat less game meat than pork.

Prevalence rates of HEV RNA in the wild boar populations of other European countries like the Netherlands (2.0%) and Germany (5.0%) are similar (*2*), and rates of HEV RNA are higher among wild boars in Hungary (12.2%) and Italy (25.0%) (*2*) and among deer in Spain (13.6%), the Netherlands (15.0%), and Hungary (34.4%) (*2*). However, despite these HEV RNA prevalence figures, no HEV-hyperendemic region in these countries has been described. Thus, the prevalence of HEV RNA among wildlife alone is not enough to explain the high seroprevalence in a specific area. Contact with the animal reservoir and local dietary practices must be taken into account.

Prevalence of HEV RNA among wild rabbits in this study (5%) is lower than that found by a previous (2010) study of wild rabbits from the Haute-Garonne Department in southwestern France (6/12; 50%) (*7*). The low prevalence rate reported here could be the result of our shorter collection time (5 months in our study vs. 3 years in the previous study). It is also possible that HEV has become less prevalent since the previous study was done. The description of a strain from humans closely related to a strain from rabbits indicates that zoonotic transmission of HEV from rabbits is possible (*7*).

A recent study has shown that rats are competent hosts for HEV3, suggesting that rodents (e.g., coypu) may be an alternative reservoir for zoonotic strain HEV3 (*14*). Coypu meat is eaten as pâté and may be an unexpected source of HEV foodborne zoonotic HEV infection. However, none of the 78 coypu livers tested were positive for HEV RNA. Thus, coypu do not seem to carry zoonotic strains of HEV. However, significant nucleotide changes may be present in the HEV strains in coypu. Thus, the primers used may have not been able to amplify HEV RNA.

In conclusion, the prevalence of HEV RNA in wild boars, deer, and rabbits is similar to that previously reported for pigs. Consumption of the meat of these wild animals, and of pig liver sausage, all contribute to the HEV epidemiology in the Midi-Pyrénées area because of specific local eating habits. Game meat from this part of France should be cooked thoroughly to minimize the risk for HEV infection (*15*).
